# Glioma Cells in the Tumor Periphery Have a Stem Cell Phenotype

**DOI:** 10.1371/journal.pone.0155106

**Published:** 2016-05-12

**Authors:** Sune Munthe, Stine Asferg Petterson, Rikke Hedegaard Dahlrot, Frantz Rom Poulsen, Steinbjørn Hansen, Bjarne Winther Kristensen

**Affiliations:** 1 Department of Pathology, Odense University Hospital, 5000 Odense C, Denmark; 2 Institute of Clinical Research, University of Southern Denmark, 5000 Odense C, Denmark; 3 Department of Neurosurgery, Odense University Hospital, 5000 Odense C, Denmark; 4 Department of Oncology, Odense University Hospital, 5000 Odense C, Denmark; University of Michigan School of Medicine, UNITED STATES

## Abstract

Gliomas are highly infiltrative tumors incurable with surgery. Although surgery removes the bulk tumor, tumor cells in the periphery are left behind resulting in tumor relapses. The aim of the present study was to characterize the phenotype of tumor cells in the periphery focusing on tumor stemness, proliferation and chemo-resistance. This was investigated in situ in patient glioma tissue as well as in orthotopic glioblastoma xenografts. We identified 26 gliomas having the R132 mutation in Isocitrate DeHydrogenase 1 (mIDH1). A double immunofluorescence approach identifying mIDH1 positive tumor cells and a panel of markers was used. The panel comprised of six stem cell-related markers (CD133, Musashi-1, Bmi-1, Sox-2, Nestin and Glut-3), a proliferation marker (Ki-67) as well as a chemo-resistance marker (MGMT). Computer-based automated classifiers were designed to measure the mIDH1 positive nucleus area-fraction of the chosen markers. Moreover, orthotopic glioblastoma xenografts from five different patient-derived spheroid cultures were obtained and the tumor cells identified by human specific immunohistochemical markers. The results showed that tumor cells in the periphery of patient gliomas expressed stem cell markers, however for most markers at a significantly lower level than in the tumor core. The Ki-67 level was slightly reduced in the periphery, whereas the MGMT level was similar. In orthotopic glioblastoma xenografts all markers showed similar levels in the core and periphery. In conclusion tumor cells in the periphery of patient gliomas have a stem cell phenotype, although it is less pronounced than in the tumor core. Novel therapies aiming at preventing recurrence should therefore take tumor stemness into account. Migrating cells in orthotopic glioblastoma xenografts preserve expression and stem cell markers. The orthotopic model therefore has a promising translational potential.

## Introduction

Treatment of gliomas is a major challenge. Despite treatment consisting of surgery, chemotherapy and radiation the mean survival of patients with the most common and malignant primary brain tumor, the WHO grade IV Glioblastoma multiforme (GBM), is approximately 14.6 months [1]. A major challenge in treatment of gliomas is the high migratory potential of glioma cells [2]. Migrating glioma cells are not eligible to surgery and therefore eradication by radiation and chemotherapy is standard strategy.

The high resistance of gliomas against conventional radiation and chemotherapy has been suggested to be due to the existence of immature cancer stem cells (CSC), which are tumor cells with a stem cell-like phenotype sustaining glioma growth through asymmetric cell division [3, 4]. These cells have been suggested to be resistant towards conventional treatment due to enhanced DNA repair and enhanced expression of ATP-binding cassette drug transporters [5]. The presence of CSCs in the periphery of gliomas has not previously been thoroughly investigated. We therefore hypothesized that migrating glioma cells display a stem cell phenotype and express stem cell markers. Since proliferation and chemo-resistance are important determinants for the effect of radiation and chemotherapy, the markers Ki-67 and MGMT were included. The aim of this study was to characterize the expression of stem cell markers as well as markers of proliferation and chemo-resistance in migrating glioma cells by using a double immunofluorescence approach on patient glioma tissue and GBM xenografts.

In patient tumor tissue a mutated form of Isocitrate dehydrogenase 1 (mIDH1) [6] was used as a tumor cell specific marker. The somatic point mutation that affects codon 132 is the most frequent IDH1 mutation and a specific well described antibody recognizing mIDH1 R132 has recently been developed [7, 8]. This mutation is primarily associated with grade II and III gliomas but is also found in secondary GBMs [9].

In glioma research the most preferred *in vivo* model is the orthotopic xenograft model. Here GBM cells are implanted into the brain of immunosuppressed animals. The model is well established in our laboratory and has been described by several groups [10, 11]. Using this model together with a double immunofluorescence approach and human specific markers to identify the tumor cells, the marker expression in migrating glioma cells was characterized. For this characterization a panel of markers similar to that used for patient gliomas was used.

We have previously used fluorescence for quantification of biomarkers in gliomas [12]. In this study we combined the tumor cell specific markers in a double immunofluorescence protocol with the following panel of stem cell markers: CD133 [4, 13–18], Nestin [18–24], Musashi-1 [18, 25–28], Sox-2 [18, 29–32] and Bmi-1 [18, 29, 32, 33], to characterize the phenotype of migrating glioma cells. We also investigated the glucose transporter type III (Glut-3) in the core and periphery since it has been reported to be associated with stemness [34]. Furthermore, we investigated the expression of the DNA repair enzyme O^6^-methylguanine-DNA methyltransferase (MGMT) in the core of the tumor and compared it to the periphery, since MGMT is a strong prognostic and predictive marker for the effect of temozolomide in the upfront GBM treatment [35]. The proliferation of migrating tumor cells has previously been investigated by Sabit *et al*. [36] who investigated 11 patients. We wanted to futher explore this in our cohort consisting of 26 patients as well as by using the orthotopic GBM xenograft model.

## Material and Methods

### Patient selection and pathology

Adult residents in the Region of Southern Denmark diagnosed with primary brain cancer between 1^st^ of January 2005 and 31^st^ of December 2009 were identified in the Danish Cancer Register (DCR). Patients with the histopathological codes for gliomas (M 94003, M 94013, M 94403, M 94503, M 94513, M 93823, and M 93853) were considered for inclusion in the present study, giving a total of 277 patients. The mIDH1 status was identified by immunohistochemical staining; 47 patients were mIDH1 positive. Twenty six patients were identified with both a tumor core and a periphery zone present in the same tumor section. The different mIDH1 positive gliomas are listed according to the WHO classification in Table 1. The histopathological evaluation was carried out at the Department of Pathology at Odense University Hospital. All tissue samples were evaluated by two pathologists and classified according to WHO guidelines 2007 [37].

**Table 1:** Summary of patients.

Histopathological diagnosis	Number of mIDH1 patients	Age (years)		WHO grade
		Median	Range	
diffuse astrocytoma	6 (23.1%)	41	30–62	II
oligodendroglioma	7 (26.9%)	48.9	31–81	II
oligoastrocytoma	5 (19.2%)	46.8	26–74	II
anaplastic astrocytoma	2 (7.7%)	46	34–58	III
anaplastic oligodendroglioma	1 (3.8%)	38	38	III
anaplastic oligoastrocytoma	2 (7.7%)	41	36–46	III
glioblastoma	3 (11.5 %)	60.3	43–75	IV
Total	26	46.7	26–81	

### Xenograft model

For establishing the orthotopic xenograft model we used 5 different GBM spheroid cultures, established in our laboratory from patient-derived GBM tumor tissue, collected at the Department of Neurosurgery, Odense University Hospital. Tumor cells were grown and stem cell phenotypes validated as previously described in our laboratory [38]. The five GBM spheroid cultures were named: T78, T86, T87, T111 and T113.

Female Balb c nu/nu mice 7–8 weeks of age were anesthetized subcutaneously with injection of a mixture of Hypnorm and Dormicum (0,1ml/10g). The mice were placed in a stereotactic frame (Model 900, David Kopf Instrument). A midline incision was made exposing bregma and a burr hole was made 1 mm anterior and 2 mm lateral to bregma. A syringe (2 μl Hamilton syringe) with a blunt needle containing 150.000 cells/μl was inserted 3 mm into the brain and 2 μl were injected slowly into the brain over several minutes. The needle was slowly removed to prevent a vacuum causing the tumor cells to escape. The skin was sutured with resorbable sutures. If the mice showed any signs of neurological deficit or weight loss more than 20%, the mice were euthanized in a carbon dioxide chamber. The brains were immediately removed and fixated in 4% formalin for 48 hours. Before paraffin embedding the brains were manually divided by 1mm coronal sections. Histological sections were afterwards cut and immunohistochemically stained with Vimentin, a human specific antibody. In mice implanted with the GBM spheroid culture T86 CD56, another human specific antibody, was used for identification of the tumor cells. The GBM spheroid cultures T86 and T113 were found to have unmethylated MGMT promoter, whereas the remaining GBM spheroid cultures were methylated in the promoter region. We therefore did not investigate the MGMT in the *in vivo* model. We defined the core in our *in vivo* model as striatum and the periphery as corpus callosum in the contra lateral side.

### Immunohistochemical staining

Histological sections of three μm were cut on a microtome and placed on glass slides. Tissue was deparaffinized, and antigen retrieval was carried out in a microwave oven using the Tris EGTA buffer. Slides were stained on the AutostainerPlus platform (DAKO, Glostrup, Denmark). For mIDH1 staining slides were incubated with antibody (mIDH1 R132H, Clone H09, Dianova, 1:100) and the detection system ultraViewTM Universal DAB Detection Kit (Ventana Medical Systems) was subsequently used. For each patient, tumor slide was stained by a double immuno-fluorescence approach combining mIDH1 and 6 different stem cell-related markers: CD133 (clone: W6B3C1, Miltenyi Biotec), Musashi-1 (clone:14H1, MBL International), Bmi-1 (clone: F6, Upstate Cell Signaling Solution), Sox-2 (clone: 245610, R&D Systems Inc.), Nestin (clone: 196908, R&D Systems Inc.), Glut-3 (clone: HPA006539, Atlas Antibodies), MGMT (clone: MT23.2, Invitrogen) and the proliferation marker Ki-67 (clone: MIB1, Beckman Coulter). The CSA II Biotin-free Tyramide signal Amplification System kit (DAKO) was used for detection of mIDH1 (1+1600). Detection of the second antibody was performed using a Tyramide Signal Amplification (TSA) Plus System with Cyanin 5 (Cy5); CD133 (1+40), Musashi (1+200), Bmi-1 (1+200), Sox-2 (1+400), Nestin (1+200), GLUT-3 (1+100), MGMT (1+100) and Ki67 (1+800). The nuclei were counterstained with 4´,6 diamidino-2-phemylindole (Dapi) (VWR Internation ApS).

For each xenograft slide a double immuno-fluorescence staining with Vimentin and the same six stem cell markers and Ki-67 was made. For xenograft slides with T86 tumors, CD56 was used as a tumor marker since it was Vimentin negative. The Alexa Flour-488 donkey anti-rabbit (1+100) was used to detect Vimentin (1+400, EP20, Epitomics) and CSA II Biotin-free Tyramide signal Amplification System kit (DAKO) was used for detection of CD56 (1+3200, CO4-NCAM, Neomarkers) in the T86 tissue samples. The nuclei were counterstained with 4´.6 diamidino-2-phemylindole (Dapi) (VWR Internation ApS).

### Automated Quantitative analysis

Super images of whole slides were taken at 1.25x magnification using a Leica DM6000 B microscope with an Olympus DP72 camera with bright field settings. Subsequently the region of interest (ROI), both the core and periphery, was manually outlined for each tumor section using the Visiopharm Integrator System (VIS) version: 4.5.6.440 (Visiopharm, Hørsholm, Denmark). Sampling was performed at 20x magnification with a minimum of 15 images in each ROI. Images were reviewed to ensure that no artifacts or blurring were present. Then images were analyzed using an algorithm developed in the Visiomorph software module. A specific classifier was developed for each double-fluorescence staining according to the Visiopharm Manual. Classifiers were designed and trained so nuclear area corresponding to DAPI staining of positive cells were measured. In each specific classifier we calculated the total area of the nuclei of tumor cells of interest (labeled by tumor cell specific marker and marker of interest) and the total area of remaining tumor cell nuclei (labeled by tumor cell specific marker but not by marker of interest). From these areas, the nuclear area fraction of tumor cells labeled by marker of interest was calculated in core and periphery. This fraction was determined based on the whole mIDH1 positive cell population in each ROI. It was not determined on a single cell basis, since core areas with very high cellularity did not allow complete separation of the DAPI stained nuclei.

### Statistics

Data was analyzed in GraphPad Prism version 5.01. The comparison of area fraction in the core and periphery was performed with an unpaired t-test. Statistical significance was defined as p < 0.05.

### Ethics

The official Danish ethical review board named the Regional Scientific Ethical Committee of the Region of Southern Demark approved the use of human glioma tissue (permission J. No. S-2011 0022) in the current study. Written informant consent was obtained from all participants. The use of animals in the present study was approved by The Animal Experiment Inspectorate in Denmark (J. Nr. 2013/15-2934-00973).

## Results

**Fig 1:**
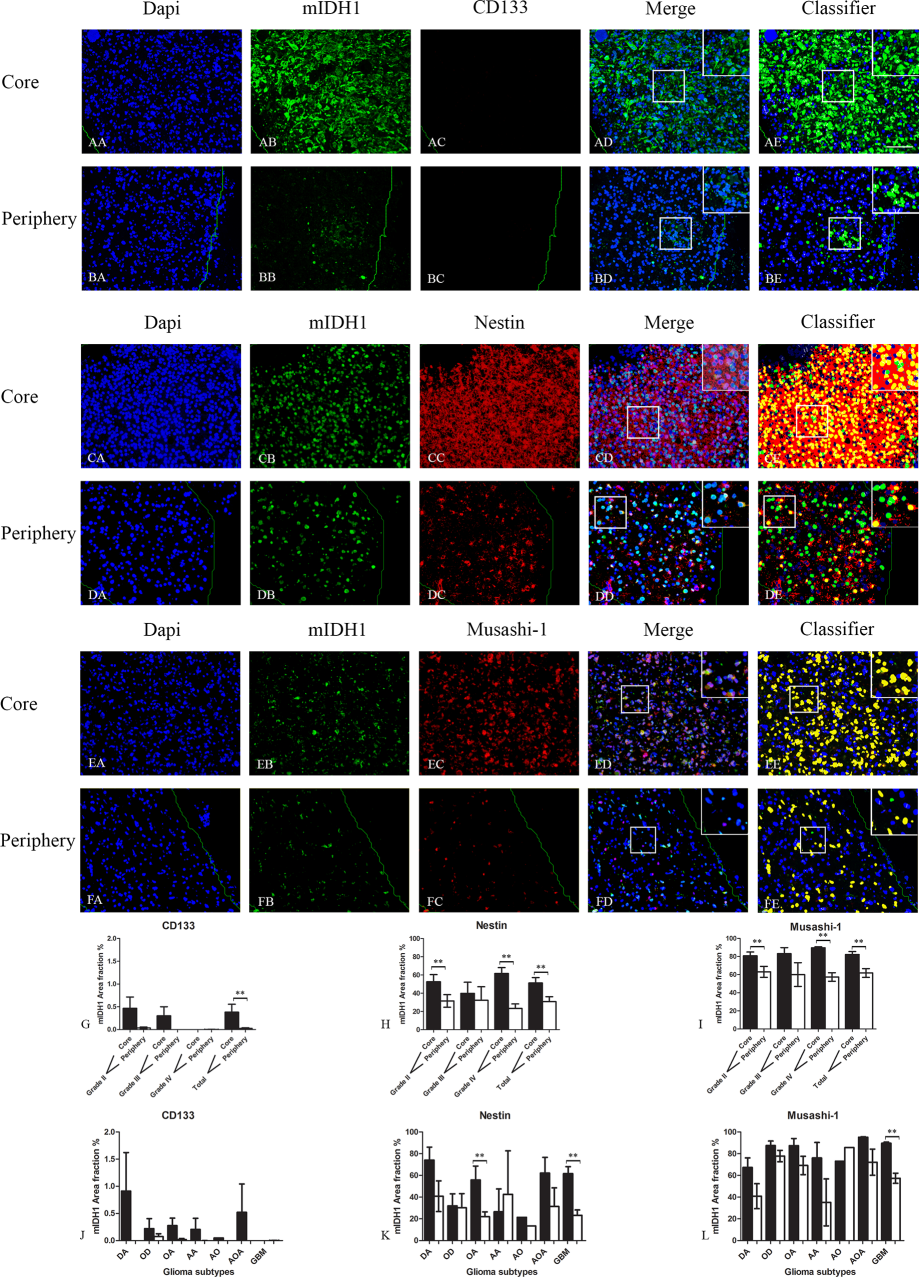
Double immunofluorescence staining of core and periphery of patient glioblastomas. Histological sections were stained with Dapi (blue), IDH1 (green) and CD133 (red) (AA-BE), Nestin (red) (CA-DE) and Musashi-1 (red) (EA to FE). The software-based classifier is shown in the right column. The classifier illustrates tumor cells co-expressing markers of interest in yellow and tumor cells not co-expressing markers of interest in green. The fluorescence stainings were quantified in both core and periphery for CD133 (G,J), Nestin (H,K) and Musashi-1 (I,L). Statistical comparison was performed using student’s t-test and ANOVA, ** p< 0.01. Scalebar: 200μm.

CD133 expression was significantly reduced in migrating tumor cells in the tumor periphery compared to tumor cells in the core region when comparing levels in all tumor samples. Similar but not significant results were obtained in the different grades and glioma subtypes (Fig 1G and 1J).

Nestin expression was significantly reduced in the periphery for tumor grade II and IV and for all gliomas together (Fig 1H). For the different glioma subtypes only Oligo-Astrocytoma (OA) and GBM revealed significantly reduced expression in the periphery although the same trend was found in the other subtypes (Fig 1K).

The expression of Musashi-1 was significantly reduced in the periphery for grade II and IV and in all gliomas together (Fig 1I). For the different subtypes of gliomas only GBM had a reduced expression in the periphery, although the same trend was found in the other subtypes (Fig 1L).

**Fig 2:**
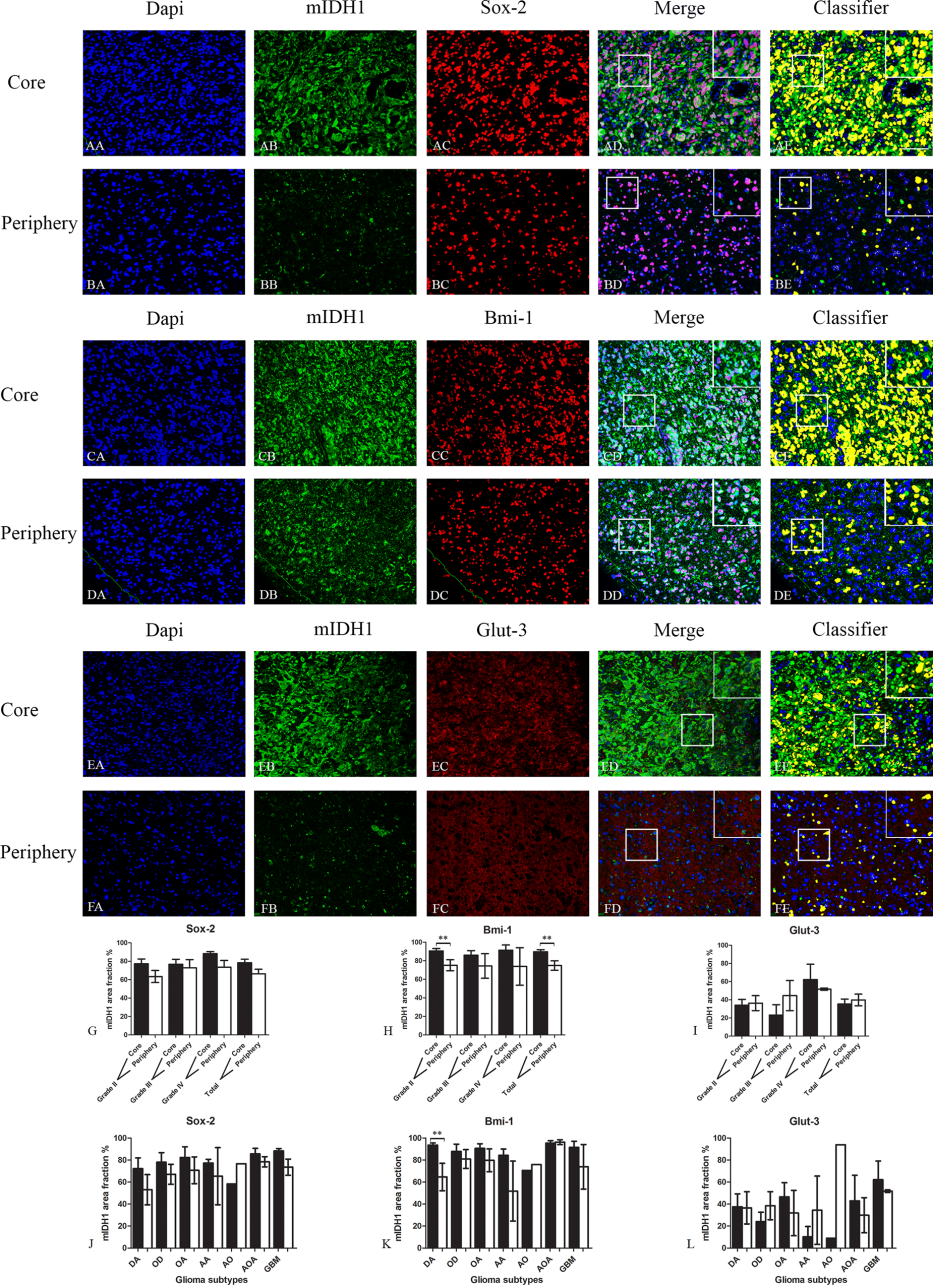
Double immunofluorescence staining of core and periphery of patient glioblastomas. Histological sections were stained with Dapi (blue), IDH1 (green) and Sox-2 (red) (AA-BE), Bmi-1 (red) (CA-DE) and Glut-3 (red) (EA to FE). The software-based classifier is shown in the right column. The classifier illustrates tumor cells co-expressing markers of interest in yellow and tumor cells not co-expressing markers of interest in green. The fluorescence stainings were quantified in both core and periphery for Sox-2 (G,J), Bmi-1 (H,K) and Glut-3 (I,L). Statistical comparison was performed using student’s t-test and ANOVA, ** p< 0.01. Scalebar: 200μm.

The expression of Sox-2 was reduced in the periphery for all tumor grades and subtypes (S1 Fig) except for the Anaplastic Oligodendroglioma (AO), but without reaching significance (Fig 2G and 2J).

The expression of Bmi-1 was significantly reduced in the periphery of grade II tumors and all gliomas together (Fig 2H). For subtypes, only the Diffuse Astrocytoma (DA) had a significantly reduced expression in the periphery. The expression of Bmi-1 was reduced in the periphery of all subtypes except the Anaplastic Oligodendroglioma (AO), but without reaching significance (Fig 2K).

Glut-3 expression was apparently expressed at similar levels in core and periphery (Fig 2I and 2L).

**Fig 3:**
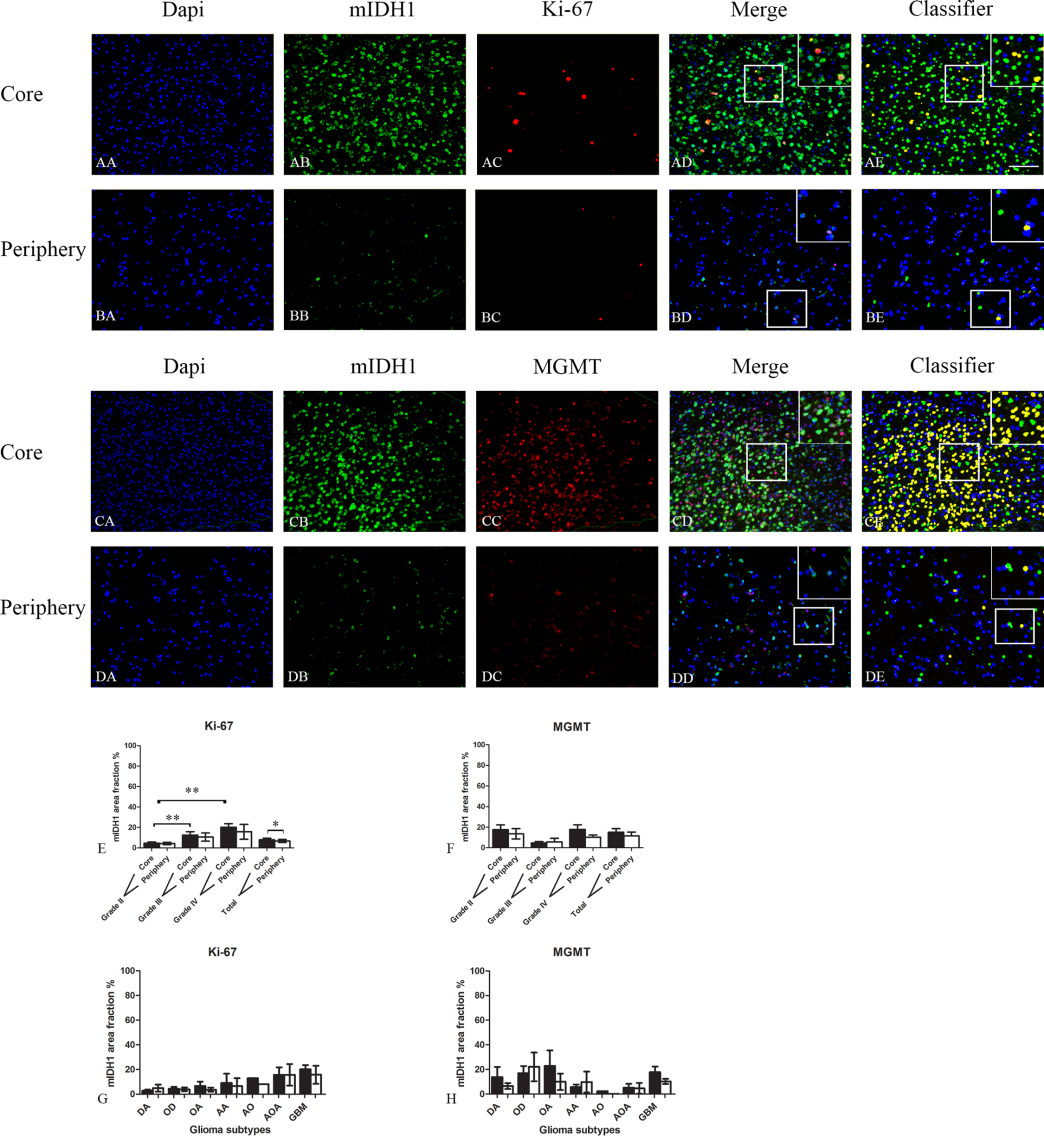
Double immunofluorescence staining of core and periphery of patient gliomas. Two glioblastomas (AA-BE and CA-DE) were stained with Dapi (blue), IDH1 (green) and Ki-67 (red) and MGMT (red). The software-based classifier is shown in the right column. The classifier illustrates tumor cells co-expressing markers of interest in yellow and tumor cells not co-expressing markers of interest in green. The fluorescence stainings were quantified in both core and periphery for Ki-67 (E,G) and MGMT (F,H). Statistical comparison was performed using student’s t-test and ANOVA, ** p< 0.01. Scalebar: 200μm.

The expression of Ki-67 was significantly reduced in the periphery for all gliomas together (Fig 3E). Similar but no significantly reduced expression of Ki-67 in periphery compared to core was obtained in the different grades and glioma subtypes (Fig 3E and 3G).

MGMT was expressed at similar levels in core and periphery (Fig 3F and 3H).

Comparing expression of the different markers between grades, we did observe a significant increase in the Ki-67 expression in the core area from grade II to grade IV (Fig 3E). There was no significant increase of expression of other markers with grade.

**Fig 4:**
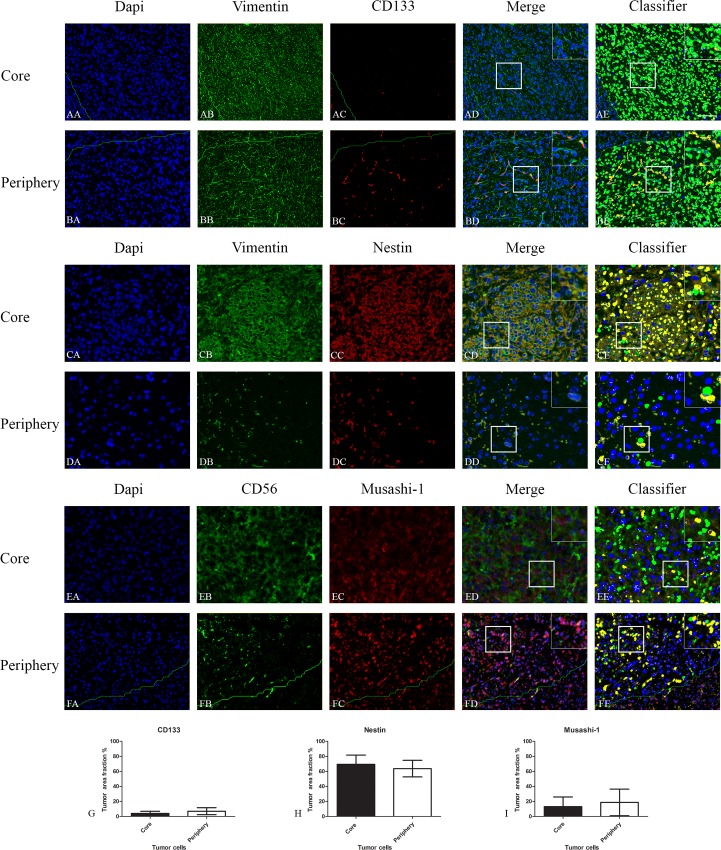
Double immunofluorescence staining of core and periphery in orthotopic model with five different patient-derived GBM spheroid cultures. Tissues were stained with Dapi (blue), Vimentin/CD56 (green) and CD133 (red), Nestin (red) and Musashi-1 (red). The software-based classifier is shown in the right column. The classifier illustrates tumor cells co-expressing markers of interest in yellow and tumor cells not co-expressing markers of interest in green. The fluorescence stainings were quantified in both core and periphery for CD133 (G), Nestin (H) and Musashi-1 (I). Statistical comparison was performed using student’s t-test. Scalebar: 200μm.

**Fig 5:**
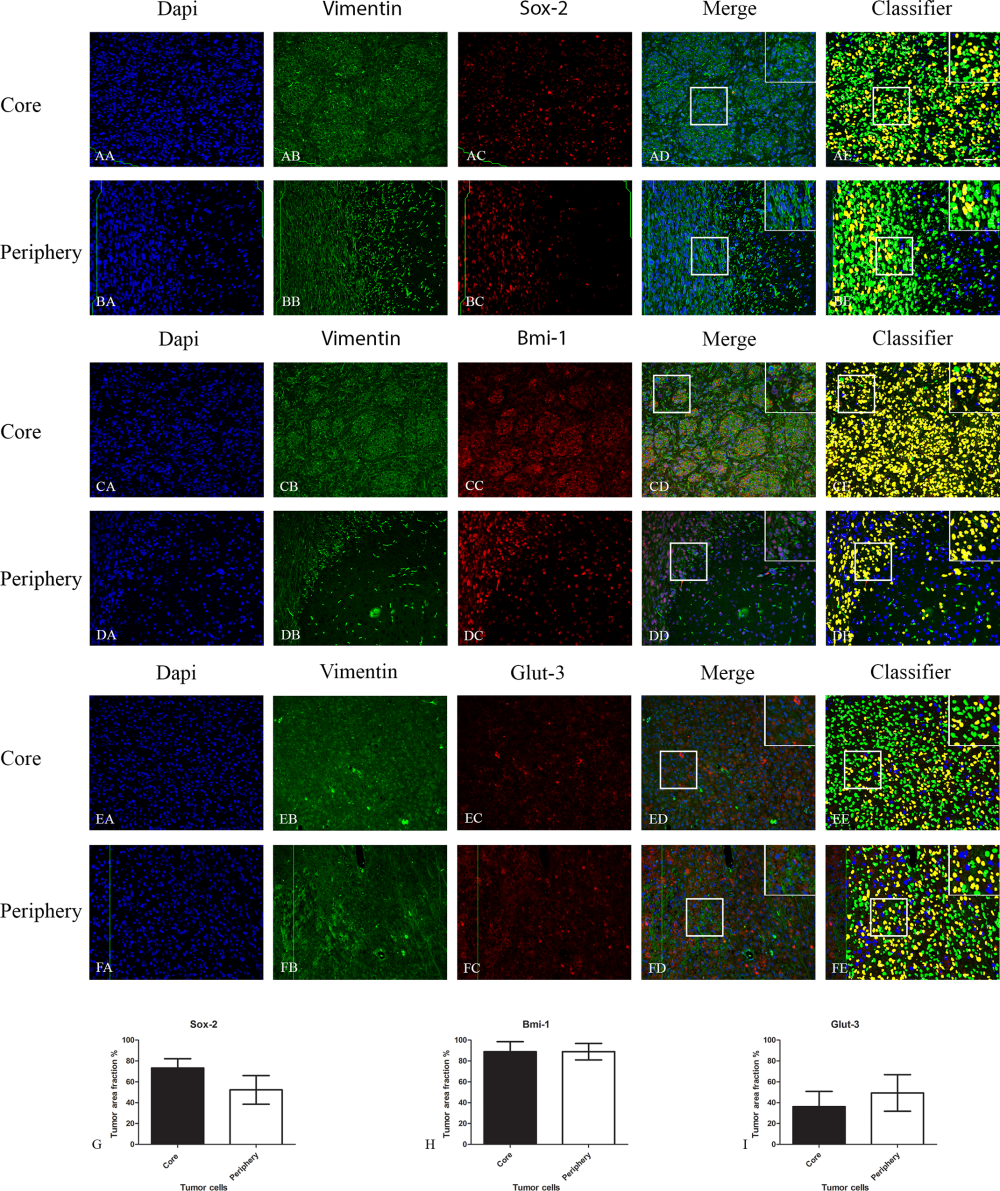
Double immunofluorescence staining of core and periphery in orthotopic model with five different patient derived GBM spheroid cultures. Tissues were stained with Dapi (blue), Vimentin (green) and Sox-2 (red), Bmi-1 (red) and Glut-3 (red). The software-based classifier is shown in the right column. The classifier illustrates tumor cells co-expressing markers of interest in yellow and tumor cells not co-expressing markers of interest in green. The fluorescence stainings were quantified in both central part and periphery for Sox-2 (G), Bmi-1 (H) and Glut-3 (I). Statistical comparison was performed using student’s t-test. Scalebar: 200μm.

**Fig 6:**
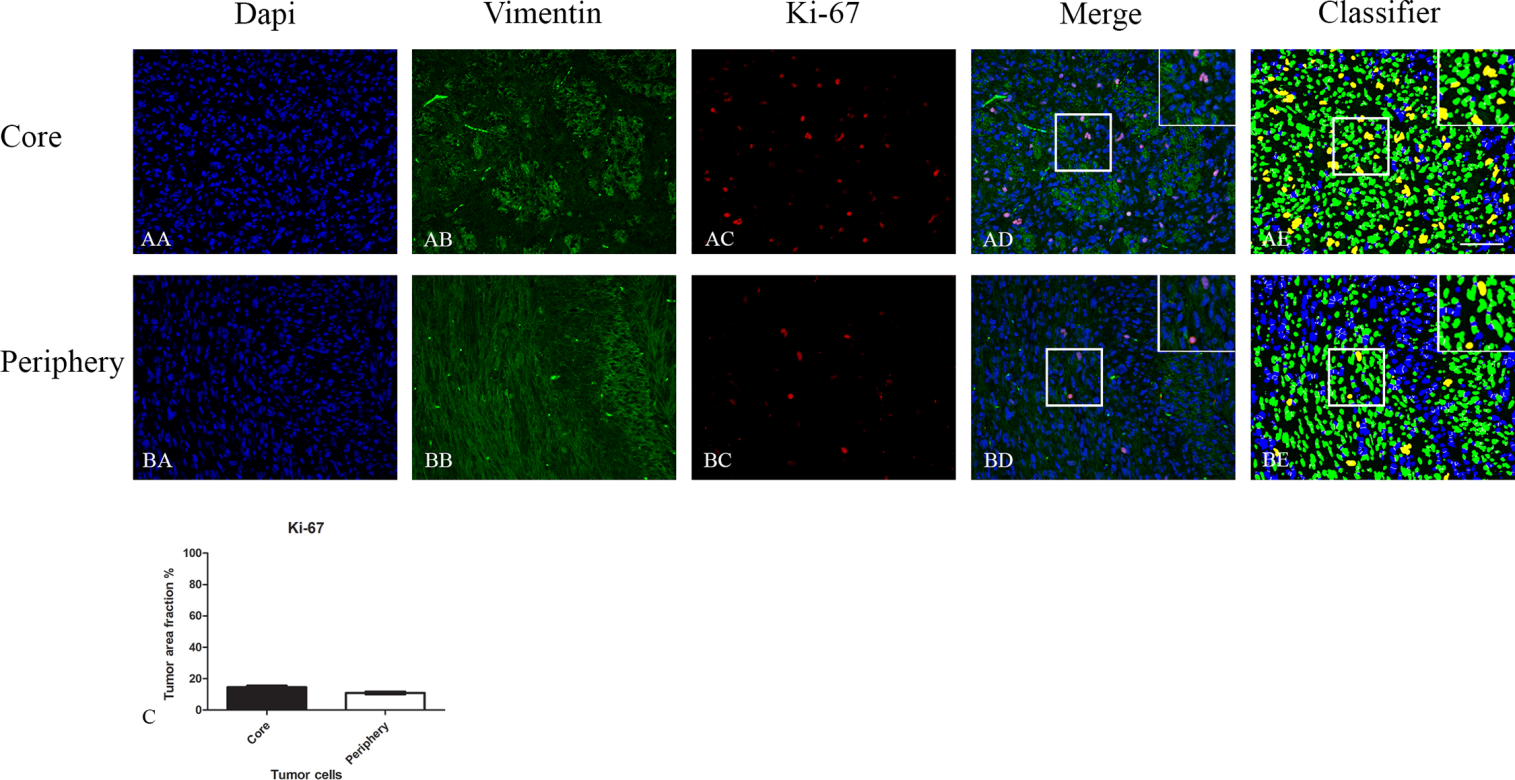
Double immunofluorescence staining of core and periphery in orthotopic model with five different patient derived GBM spheroid cultures. Tissues were stained with Dapi (blue), Vimentin (green) and Ki-67 (red). The software-based classifier is shown in the right column. The classifier illustrates tumor cells co-expressing markers of interest in yellow and tumor cells not co-expressing markers of interest in green. The fluorescence stainings were quantified in both core and periphery for Ki-67 (C). Statistical comparison was performed using student’s t-test. Scalebar: 200μm.

In the GBM xenografts the different markers were expressed at similar levels in core and periphery (Figs 4, 5 and 6).

## Discussion

Our results suggest that tumor cells in the periphery express CSC-markers both in patient gliomas and in orthotopic xenografts. The CSC hypothesis states that tumor growth is driven by a subpopulation of tumorigenic CSCs [39]. Huang *et al*. and Bao *et al*. showed that CSCs in GBMs were extremely resistant to conventional radiation and chemotherapies [3, 40]. Together these results suggest that the CSC hypothesis also extend to tumor cells left in the periphery after surgery, although the vast majority of studies have been performed on removed central tumor material. The presence of CSCs in the periphery in all glioma grades and sub-types indicates that these CSCs could be the reason for treatment failure and recurrence in glioma patients. Extending the CSC hypothesis to tumor cells left in the periphery after surgery for all gliomas is fully in line with the well know observation that macro radical resection of the tumors increases survival compared to biopsies and suboptimal resection [41, 42].

Expression of CD133 was found at lower level compared to the other stem cell markers. This observation corresponds to what has been shown in other studies [43]. We have previously shown that it is of great importance which CD133 clone is used; e.g. whether the CD133 clone targets the non-glycosylated epitope (i.e. the C24B9 clone) or the glycosylated extracellular epitope (i.e. the AC133 or W6B3C1)[44]. In this study we used the W6B3C1 clone, which we previously have identified as an antibody clone labeling both membrane and cytoplasm of tumor cells [44–46]. Most importantly this clone has been shown to be associated with asymmetric tumor cell division, which is a hallmark of cancer stem cells [44, 47].

For Ki-67, the expression was slightly reduced in the periphery for all gliomas together. Comparing the expression between grades, we did however observe a significant increase in the Ki-67 expression in the core area from grade II to grade IV (Fig 3E). There was no significant increase in expression of other markers together with tumor grade. This is in line with the results from Sabit *et al*. [36] who used a similar mIDH1 double fluorescence approach (11 patients). However Sabit *et al*. did not use a computer-based algorithm, but two independent neuropathologists to evaluate the tumor samples. The slightly reduced proliferation in the periphery suggest that both temozolomide and radiation therapy is less efficient in these areas compared to central tumor areas. This may explain why Ki-67 labeling indices found and used in prognostic studies, where tumor bulk is removed, not have shown to have a prognostic value predicted [48, 49]. In most patients, it is in fact the tumor periphery left in the patients, which receives temozolomide and radiation therapy.

The Ki-67 labeling index correlated with survival should ideally be the index obtained from the periphery.

For MGMT a similar distribution in the core and periphery was found for all tumor grades and subtypes. This means that resistance to temozolomide was preserved in migrating tumor cells. Strategies aiming at reducing chemoresistance by compromising the MGMT enzyme are therefore highly relevant for migrating glioma cells and new drugs added to temozolomide should also reach these tumor cells to be efficient.

In the GBM xenograft mouse model the results showed a similar expression of stem cell markers in core and periphery for all stem cell markers. Before implantation in mice brains, the spheroid cultures were grown in neural stem cell medium, which favor a more stem cell like phenotype. This might explain the preservation of the stem cell phenotype in migrating xenograft tumor cells compared to the reduced expression of stem cell markers found in migrating glioma cells in patients. However, to a large extend the overall levels in tumor core in patients and mice were similar, except for musashi-1 and sox-2 which were expressed at much higher or higher levels in both core and periphery in patients GBMs compared to GBM xenografts. Reasons for this may be differences e.g. in tumor microenvironment. Hypoxia is pronounced in patient high grade gliomas and known to increase expression of stem cell markers [50–52], but necrosis is not found in our GBM xenografts suggesting that this micro-environmental stimulus is less pronounced patients. Another explanation of differences between results obtained in patient tumors versus GBM xenografts is IDH1 status. The patient tumors included in this study were all IDH1 mutated whereas all GBM xenografts were obtained from IDH1 wild type GBMs. Accordingly, CD133 has been found to be expressed at higher levels in IDH1 wild type GBMs compared to IDH1 mutated GBMs [53]. However, another marker of tumor aggressiveness–Ki-67 appeared to be expressed at similar levels in patient GBMs and GBM xenografts. The expression of Ki-67 in the GBM xenograft model showed a slightly reduced but not significant reduction in the periphery (n = 5), whereas the slightly reduced level was significant in patient GBMs (n = 26). In general, our results indicate that the orthotopic model to some degree mimics the migration scenario seen in patients Studies focusing on characteristics of migrating glioma cells and how they should be targeted could therefore to some extend be done in the orthotopic model.

In conclusion tumor cells in the periphery of patient gliomas have a stem cell phenotype, although it is less pronounced than in the tumor core. Novel therapies aiming at preventing recurrence should therefore take tumor stemness into account. In addition, known resistance factors like MGMT also seem to be preserved in the periphery of patient gliomas. Migrating cells in orthotopic glioblastoma xenografts preserve expression and stem cell markers. The orthotopic model therefore has a promising translational potential.

## Supporting Information

S1 Fig Double immunofluorescence staining of core and periphery of patient gliomas. Diffuse astrocytoma (AA-BE), oligo-astrocytoma (CA-DE), oligodendroglioma (EA-FE), anaplastic astrocytoma (GA-HE) and anaplastic oligo-astrocytoma (IA-JE) were stained with Dapi (blue), IDH1 (green) and Sox-2 (red). The software-based classifier is shown in the right column. The classifier illustrates tumor cells co-expressing Sox-2 in yellow and tumor cells not co-expressing Sox-2 in green. Scalebar: 200μm.
(TIF)
