# Supplementary figures and images for: Glioma Cells in the Tumor Periphery Have a Stem Cell Phenotype
Source: PLoS One. 2016 May 12;11(5):e0155106. doi: 10.1371/journal.pone.0155106 (PMC4865242; doi:10.1371/journal.pone.0155106)

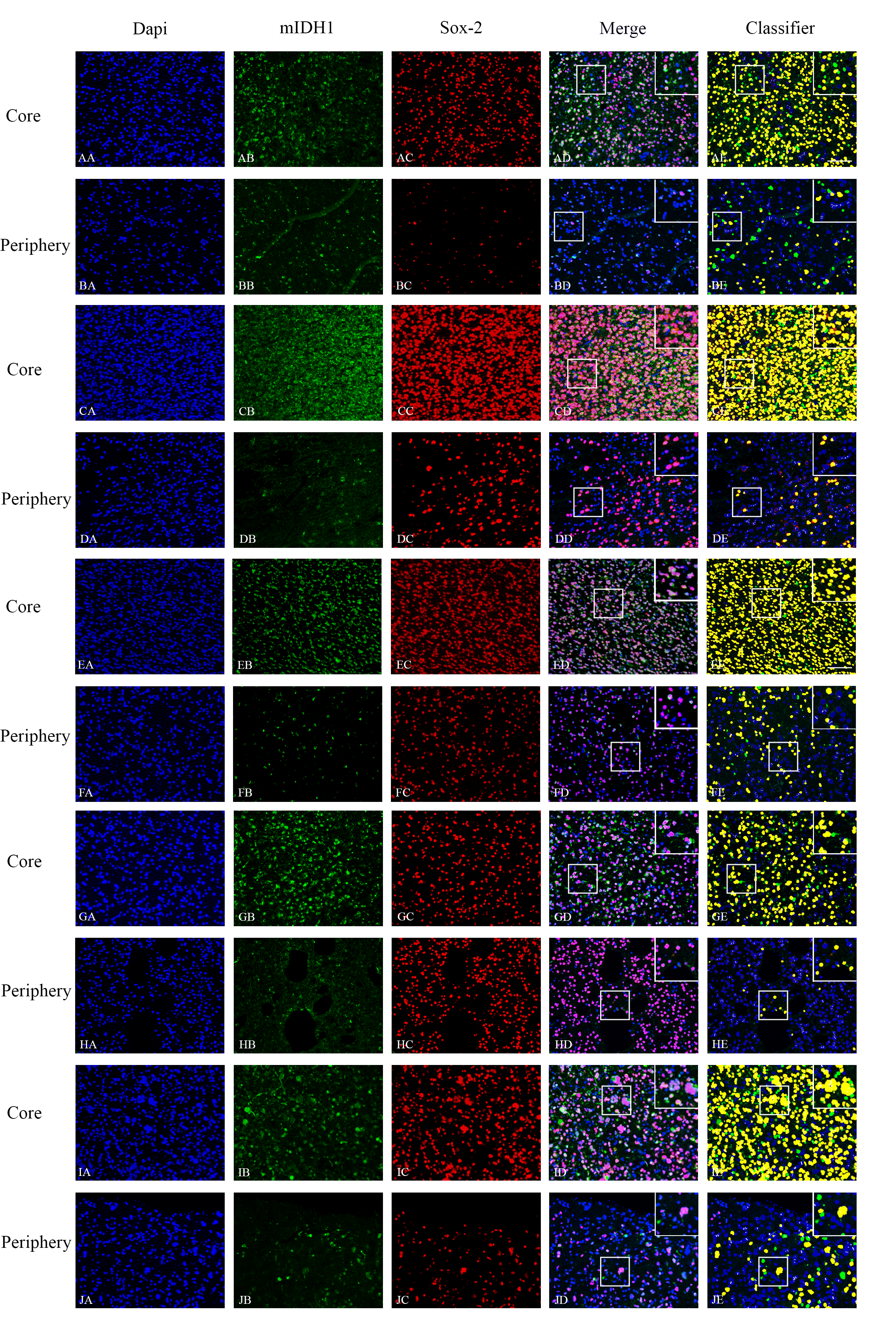

Supplement: S1 Fig — Diffuse astrocytoma (AA-BE), oligo-astrocytoma (CA-DE), oligodendroglioma (EA-FE), anaplastic astrocytoma (GA-HE) and anaplastic oligo-astrocytoma (IA-JE) were stained with Dapi (blue), IDH1 (green) and Sox-2 (red). The software-based classifier is shown in the right column. The classifier illustrates tumor cells co-expressing Sox-2 in yellow and tumor cells not co-expressing Sox-2 in green. Scalebar: 200μm. (TIF) [file pone.0155106.s001.tif]
